# Comparative studies of X chromosomes in Cervidae family

**DOI:** 10.1038/s41598-023-39088-4

**Published:** 2023-07-25

**Authors:** Anastasia A. Proskuryakova, Ekaterina S. Ivanova, Alexey I. Makunin, Denis M. Larkin, Malcolm A. Ferguson-Smith, Fengtang Yang, Olga V. Uphyrkina, Polina L. Perelman, Alexander S. Graphodatsky

**Affiliations:** 1 Institute of Molecular and Cellular Biology, SB RAS, Lavrentiev Ave 8/2, Novosibirsk, Russia 630090; 2 Novosibirsk State University, Pirogova Str. 1, Novosibirsk, Russia 630090; 3 The Royal Veterinary College, Royal College Street, University of London, London, NW1 0TU UK; 4 Department of Veterinary Medicine, Cambridge Resource Center for Comparative Genomics, University of Cambridge, Cambridge, UK; 5 School of Life Sciences and Medicine, Shandong University of Technology, Zibo, China; 6 Federal Research Center for Biodiversity of the Terrestrial Biota of East Asia, Vladivostok, Russia

**Keywords:** Evolution, Genetics

## Abstract

The family Cervidae is the second most diverse in the infraorder Pecora and is characterized by variability in the diploid chromosome numbers among species. X chromosomes in Cervidae evolved through complex chromosomal rearrangements of conserved segments within the chromosome, changes in centromere position, heterochromatic variation, and X-autosomal translocations. The family Cervidae consists of two subfamilies: Cervinae and Capreolinae. Here we build a detailed X chromosome map with 29 cattle bacterial artificial chromosomes of representatives of both subfamilies: reindeer (*Rangifer tarandus*), gray brocket deer (*Mazama gouazoubira*), Chinese water deer (*Hydropotes inermis*) (Capreolinae); black muntjac (*Muntiacus crinifron*s), tufted deer (*Elaphodus cephalophus*), sika deer (*Cervus nippon*) and red deer (*Cervus elaphus*) (Cervinae). To track chromosomal rearrangements during Cervidae evolution, we summarized new data, and compared them with available X chromosomal maps and chromosome level assemblies of other species. We demonstrate the types of rearrangements that may have underlined the variability of Cervidae X chromosomes. We detected two types of cervine X chromosome—acrocentric and submetacentric. The acrocentric type is found in three independent deer lineages (subfamily Cervinae and in two Capreolinae tribes—Odocoileini and Capreolini). We show that chromosomal rearrangements on the X-chromosome in Cervidae occur at a higher frequency than in the entire Ruminantia lineage: the rate of rearrangements is 2 per 10 million years.

## Introduction

Order Artiodactyla^1^ (recent Cetartiodactyla) is a large mammalian order that includes camels, whales, pigs, hippos, and ruminants (the suborder of animals with divided stomachs). The family Cervidae is the second most diverse in the suborder Ruminantia^2^. The systematic relationships of ruminants remain controversial. In supplementary Fig. 1 ruminants phylogeny is present in according to deep investigation of Cervidae species^3^. Cervidae includes two subfamilies: Cervinae and Capreolinae^3^. Representatives of the family are widespread in America and Eurasia and have high economic (farm animals, hunting) and ecosystem values (food source for carnivores, impact on vegetation). For decades the systematic relationships of Cervidae have been a hotly debated topic. Recent studies, based on sequences of the complete mitochondrial genome^4^ and on all available data on 318 existing and extinct species^5^, significantly clarified not only Cervidae but also artiodactyl phylogeny. There are also studies integrating molecular, morphological, and bioinformatics approaches^3^. Previously the family was divided into three subfamilies Cervinae, Capreolinae, and Hydropotinae. Recent phylogenetic data place *Hydropotes inermis*, a monotypic Hydropotinae species, in the subfamily Capreolinae^3^. Now, the subfamily Capreolinae is divided into four tribes: Capreolini, Alceini, Odocoileini, Rangiferini, and the subfamily Cervinae into two tribes: Cervini, Muntiacini^3^. In recent phylogenic research based on whole genome sequencing analysis, the status of the Muntiacini tribe was upgraded to subfamily^6^.

The accumulated cytogenetic data for the Cervidae family allows us to trace the evolution of karyotypes. Cervidae karyotypes are characterized by diversity in the diploid chromosome number (2n = 6–70)^7, 8^ and have evolved by tandem and Robertsonian translocations of acrocentric chromosomes^9^, also involving sex chromosomes. Comparative chromosome painting with whole chromosome painting probes has been employed in several studies^10–16^. These studies showed that artiodactyl autosomes evolved through fissions, fusions, and inversions. Recent research has shown the undervalued contribution of intrachromosomal rearrangements and identified evolutionary breakpoint regions not only in Cervidae^16^ but in ruminant genome evolution^16, 17^.

In most eutherian orders, only autosomal syntenic segments undergo reshuffling while the X chromosome remains highly conserved, as shown by cross-species chromosome painting^18^ and G-banding^19^. Contrarily, the X chromosome in Artiodactyla and Ruminantia demonstrates a high level of evolutionary rearrangements shown by G-banding^19^ and molecular cytogenetics studies^15, 20–23^. X-chromosomal changes in Ruminantia include inversions, centromere shifts, heterochromatic variation, and X-autosomal translocations. Recently the X chromosome evolution in different representatives of artiodactyl species was studied by high-resolution BAC (Bacterial Artificial Chromosomes) mapping^15, 20, 22–24^. Cervidae X chromosomes were investigated previously by band-specific probes^25^, BACs^15, 20, 22, 24^, and oligo^26^ probe localization. A substantial part of the cervid X chromosome can be formed by heterochromatin which can be interspersed or present in blocks in the intercalary or pericentromeric regions. The centromeric heterochromatin is mostly composed of satellite DNAs that are also present in centromeres of autosomes and gonosomes^27–29^. In Cervidae, the increased size of gonosomes caused by intercalary and pericentromeric heterochromatin blocks has been observed in reindeer^25, 27^*,* tufted deer^10^, and Indian muntjac^30^. In addition, interspecific variation of the X chromosome provides a supplemental source of phylogenetic information in the form of evolutionary rearrangements as cytogenetic markers.

There is substantial data on chromosome-level genome assemblies of Cervidae species^26, 31–34^. But not all species with a chromosome-level assembly have an assembled X chromosome^26^. In the present study, we extend the list of four species studied by detailed X chromosome BAC mapping to include species from both subfamilies and four tribes: Capreolinae—reindeer (*Rangifer tarandus,* Rangiferini), gray brocket deer (*Mazama gouazoubira,* Odocoileini), Chinese water deer (*Hydropotes inermis,* Capreolini); Cervinae—black muntjac (*Muntiacus crinifron*s, Muntiacini), tufted deer (*Elaphodus cephalophus,* Muntiacini), sika deer (*Cervus nippon,* Cervini), and red deer (*Cervus elaphus,* Cervini). We analyze previously established X chromosome assemblies and compare them with the cattle genome. We reveal chromosomal rearrangements that occur on the X chromosome in the Cervidae family. Moreover, we summarize new and previously established data and describe the fine picture of rearrangements on the cervid X chromosome in an evolutionary context.

## Results

### BAC mapping of the X chromosome in Cervidae

**Figure 1:**
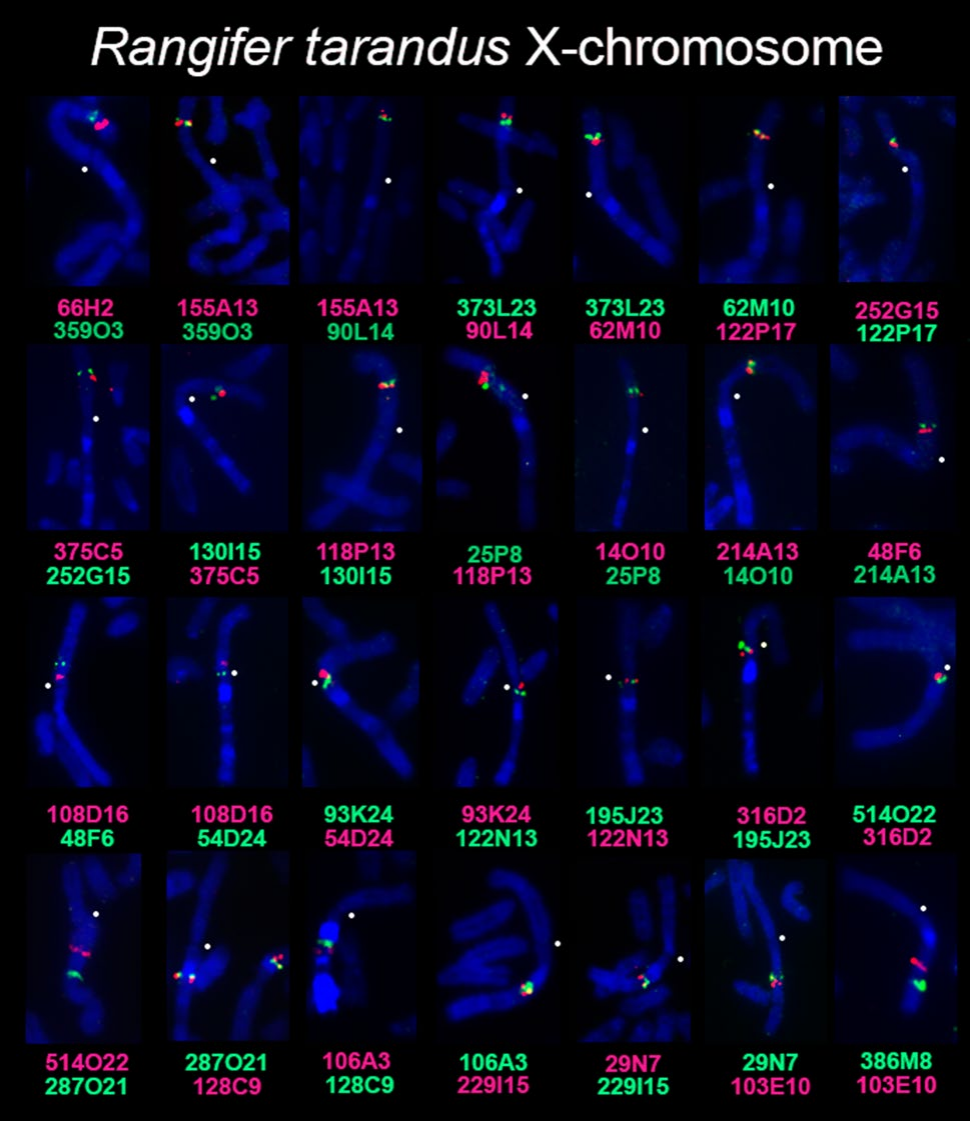
A set of 29 cattle bacterial artificial chromosome clones (BAC, CHORI-240) located on reindeer (*Rangifer tarandus*) X chromosome. Centromere positions are designated by a white dot.

In Cervidae family, an elevated level of morphological variation of the X chromosome was revealed using GTG staining^19^ and confirmed by BAC-clone hybridization in several cervid species^20, 22, 24^. To investigate the order of conserved syntenic segments on X chromosomes in the Cervidae family, 29 bovine BAC-clones were localized using FISH (fluorescence in situ hybridization) on X chromosomes of seven species from both subfamilies: black muntjak, tufted deer, sika deer, red deer, reindeer, Chinese water deer, and gray brocket deer in a series of pairwise FISH experiments. An example of the localization of BAC clones on reindeer chromosomes is presented in Fig. 1. All FISH data are presented in supplementary materials (Suppl. 2).

**Figure 2:**
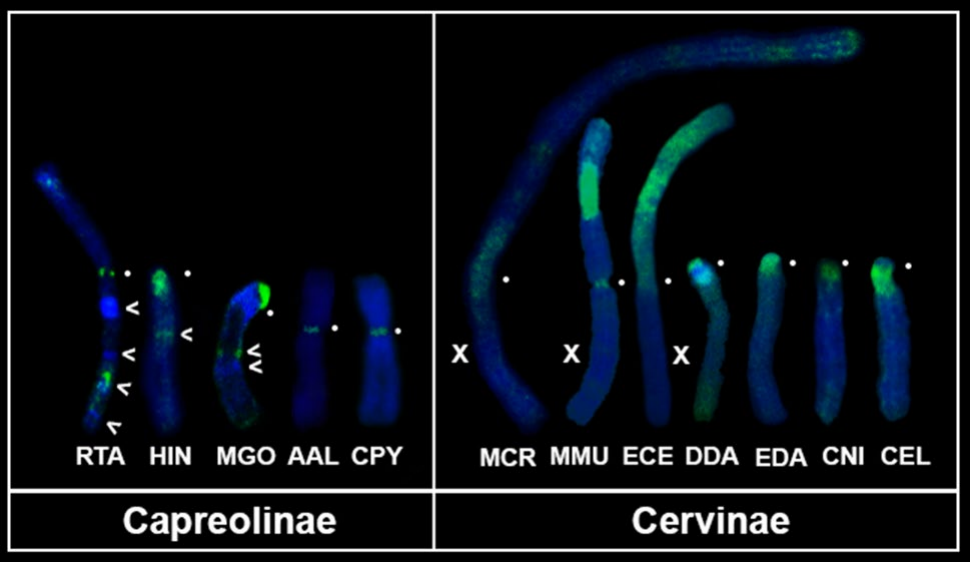
Heterochromatin on X chromosome in Cervidae species revealed by chromomycin A3-DAPI after G-banding (CDAG)^35^ staining (AT-enriched (blue) and GC-enriched (green)): reindeer (RTA), Chinese water deer (HIN), gray brocket deer (MGO), Eurasian elk (AAL), Siberian roe deer (CPY), black muntjac (MCR), Indian muntjac (MMU)^30^, tufted deer (ECE), milu (EDA), fallow deer (DDA), sika deer (CNI) and red deer (CEL). Centromere positions are designated by a white circle, blocks of interstitial heterochromatin by arrows.

The X chromosomes in Cervidae tend to accumulate the heterochromatin. For visualization of heterochromatin blocks on X-chromosomes of reindeer, Chinese water deer, gray brocket deer, Eurasian elk (*Alces alces*), Siberian roe deer (*Capreolus pygargus*), black muntjac, tufted deer, milu deer (*Elaphurus davidianus*), fallow deer (*Dama dama)*, sika deer and red deer) we performed the Combined Method of Heterogeneous Heterochromatin Detection (CDAG)^35^ (Fig. 2). In Cervinae species we observe only pericentromeric heterochromatin, whereas in Capreolinae species we detecte interstitial heterochromatic blocks not only on the reindeer X chromosome (published previously^25, 27^) but on Chinese water deer and gray brocket deer X chromosomes. Remarkably, an autosome to X chromosome translocation was previously identified in tufted deer^10^. We compared G-banded and CDAG-stained chromosomes from this study and those published previously^4^. The individual studied here does not have this translocation, which may indicate an intraspecific chromosome polymorphism for this rearrangement^36^.

**Figure 3:**
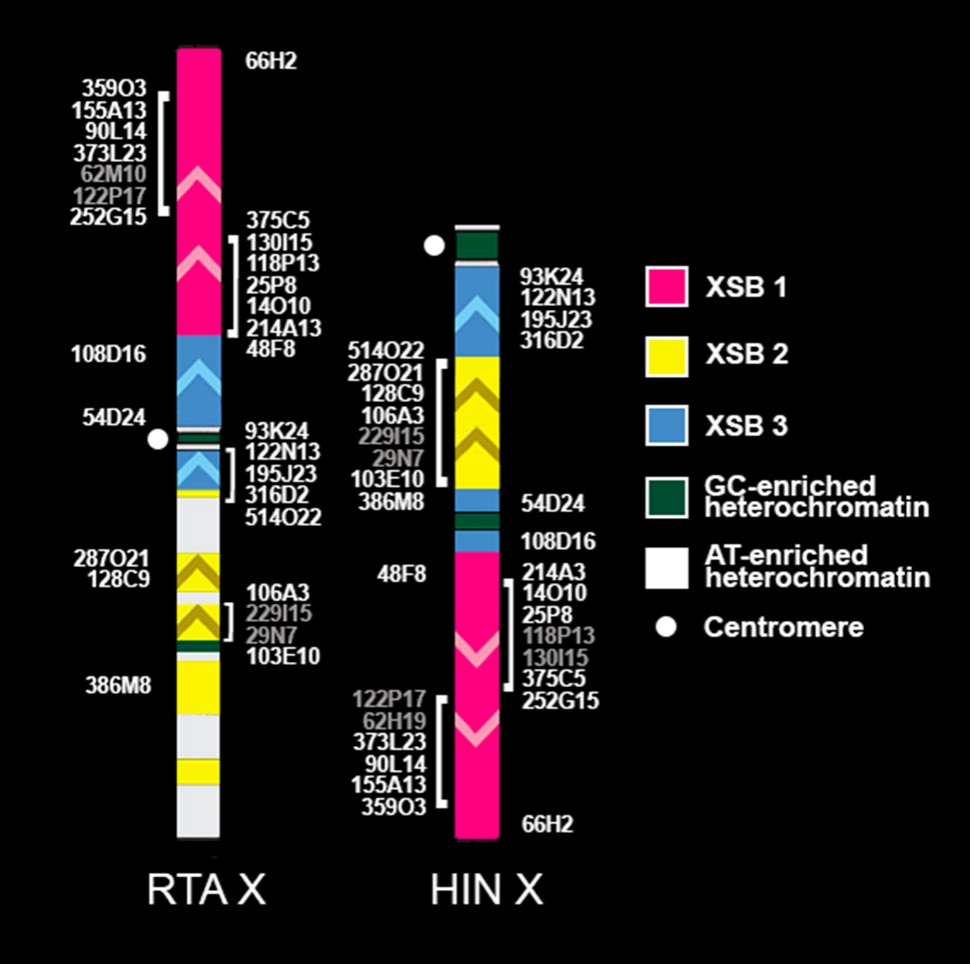
Two types of cervid X (submetacentric and acrocentric) and the order of three syntenic blocks and cattle bacterial artificial chromosome clones (BAC, CHORI-240) on reindeer (RTA X) and Chinese water deer (HIN X) X chromosomes are shown as an example. Heterochromatin blocks in reindeer are shown according to previous research^25^ and new CDAG data. The order of BACs whose names are in gray is not definitive due to close positioning at the same locus.

In total, comparative analysis of BACs’ order reveal thee syntenic blocks: X Syntenic Block 1 (15 BACs, XSB1, pink), X Syntenic Block 2 (8 BACs, XSB2, yellow), and X Syntenic Block 3 (6 BACs, XSB3, blue)^20^. The order of the BACs and syntenic blocks was detected for all investigated species. We identify two types of cervid X, that correspond to the morphology of chromosome—submetacentric and acrocentric (Fig. 3). The submetacentric type is found only in Capreolinae species (Siberian roe deer^20^, roe deer^22^, Eurasian elk^20^, reindeer). The variation of the X chromosome of these species was conditioned by centromere shift and heterochromatin expansion. Whereas the acrocentric type is present in both subfamilies: Chinese water deer, gray brocket deer (Capreolinae), black muntjac, tufted deer, sika deer, red deer, fallow deer^20^, milu deer^22^ (Cervinae). We identified an error in BACs order in blue and yellow conservative segments on the milu deer X chromosome in our previous publication^20^. The analysis of the corrected order with other research^22^ and new data shows that, milu deer X chromosome represents the typical acrocentric type of cervid X. We also compared BACs’ order from previous research^24^ of gray brocket deer X chromosome with new data. The variation of the acrocentric type of X is conditioned by autosomal translocation and heterochromatin expansion.

### Comparative analysis of cervid X chromosome assemblies

**Figure 4:**
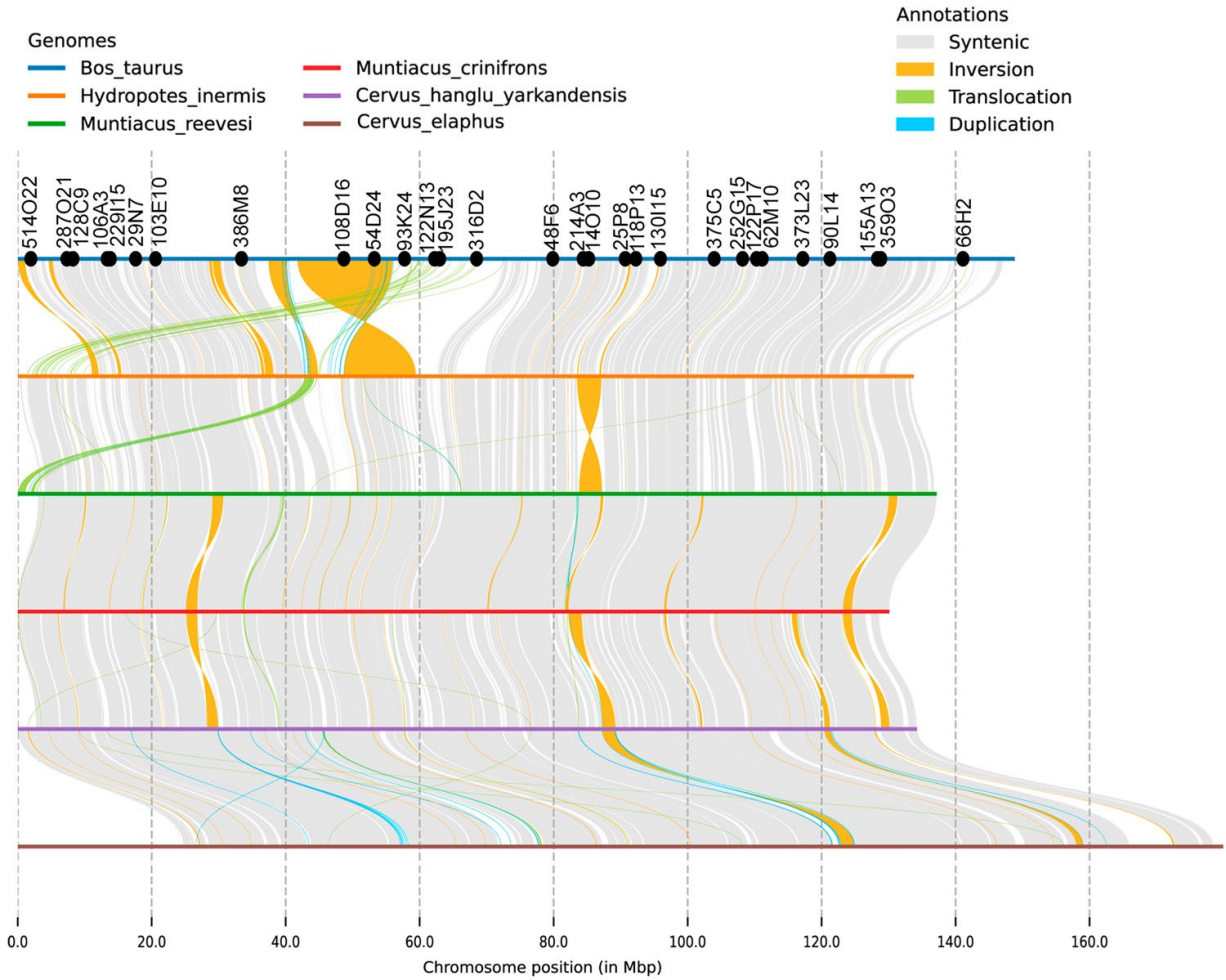
The alignment of the X chromosome in six artiodactyl species: cattle (*Bos taurus*)^37^, Chinese water deer (*Hydropotes inermis*)^32^, Chinese muntjac (*Muntiacus reevesi*)^33^, black muntjac (*Muntiacus crinifrons*)^32^, red deer (*Cervus elaphus*)^31^, Yarkand deer (*Cervus hanglu yarkandensis*)^34^. Cattle bacterial artificial chromosome clones (BAC, CHORI-240) are designated by black dots on the cattle X chromosome. The X chromosome centromeres correspond to the leftmost position for five species. The cattle X chromosome centromere is on the right of BAC 386M8. BACs’ positions in the bovine genome are listed in table (Suppl. 3).

To perform analysis of chromosome-level assemblies of X, we used available material from GenBank NCBI. We performed an alignment of the whole X chromosome assemblies of cattle^37^ and five deer species: Chinese water deer (*Hydropotes inermis*)^32^, Chinese muntjac (*Muntiacus reevesi*)^33^, black muntjac (*Muntiacus crinifrons*)^32^, red deer (*Cervus elaphus*)^31^, Yarkand deer (*Cervus hanglu yarkandensis*)^34^ (Fig. 4).

All five assembled cervid X chromosomes belong to the acrocentric type. The analysis of X chromosome alignments reveals several rearrangements that span large genomic areas as well as multiple micro rearrangements (Fig. 4). Some rearrangements include genomic regions corresponding to the BACs from the set mapped here by FISH to the X chromosomes. Major rearrangements that include mapped BACs are a translocation, several inversions and duplications. In particular relative to the cattle X chromosome, there is a translocation of the region encompassing BACs from 93K24 to 316D2 and inversion of the large region that includes BACs 108D16 and 54D24. The presence of these two rearrangements is in concordance with maps constructed by FISH on Chinese water deer and black muntjac X chromosomes (Fig. 3, Suppl. 2). Additionally, in the Chinese muntjac X chromosome we detected prominent inversions between 386M8/108D16 and 29N7/386M8. In Chinese muntjac translocation between 386M8 and 108D16 to pericentromeric region, whereas in black muntjac two inversions occurred between 29N7 and 386M8 BACs’ and in subtelomeric region were identified. The areas of the translocation and two inversions were not covered by the set of BACs. The quality of genome assemblies varies greatly, so the rearrangements identified here by genome comparison are not certain and need validation in further research, for example, by BAC localization.

Some smaller rearrangements are detected by comparative analysis of the alignment. We can see multiple microduplications (blue color) on the Cervidae X chromosome, and also multiple micro inversions (orange) in several species. An in-depth study is required to show whether these micro rearrangements represent real inversions and duplications. Maybe some, or all, are assembly or program artifacts that vary greatly in quality.

## Discussion

**Figure 5:**
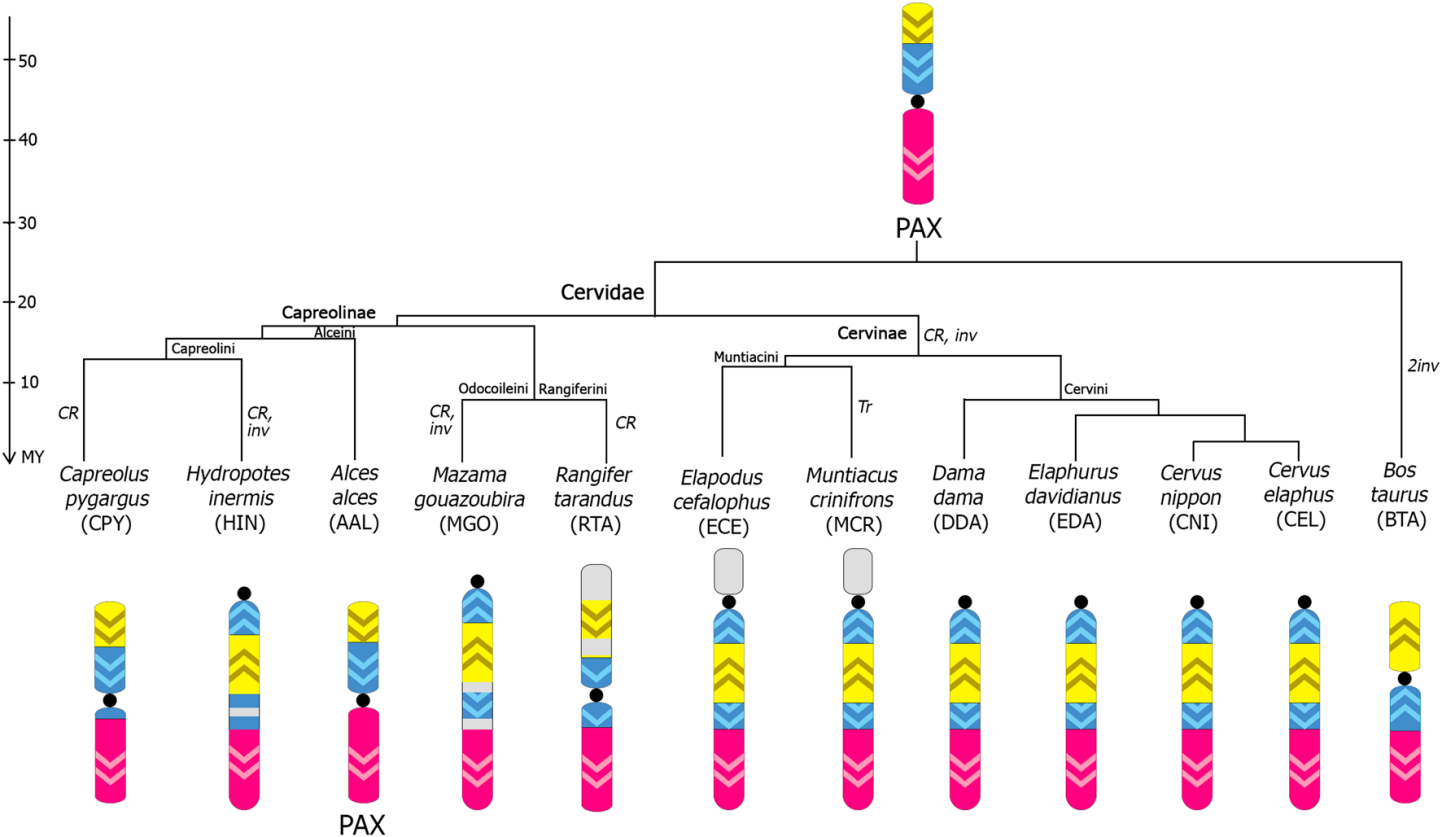
Evolutionary changes in the structure of the Cervidae X chromosome are depicted on the phylogenetic tree of the family (the tree topology is from^5^). PAX is the Pecoran ancestral X chromosome^20^. Major conservative segments of artiodactyl X are shown in pink (XSB 1), yellow (XSB 2), and blue (XSB 3). Centromere positions are designated by a black spot. Arrows show the orientation of the conservative segments. Chromosome changes are shown on the phylogenetic tree near their respective branches: CR—centromere reposition, Inv—inversion, Tr—translocation. The timescale is in million years (MY) of evolution. Tufted deer (ECE), black muntjac (MCR), and reindeer (RTA) X chromosomes are shown with an X-autosomal translocation (green block) or a heterochromatic expansion (gray block). Reindeer (RTA) X chromosome presented in an inverted position (with q arm on top).

Artiodactyl sex chromosomes, especially the X, demonstrate high levels of evolutionary rearrangements, including inversions, centromere shift, heterochromatic variation, and X-autosomal translocations^19^. Recently, a series of investigations of X chromosome evolution in different artiodactyl species, including several Cervidae species, were published employing high-resolution BAC mapping^15, 20, 22, 24^. Combining new data with previously obtained data^15, 20, 22, 24^ is possible to trace the path of transformation of the X chromosome in various branches of the Cervidae family. Figure 5 illustrates the transformation of the Cervidae X chromosome.

The assembled data show that the X chromosome of Cervinae is more conserved than the X chromosome of Capreolinae. Interestingly, the data demonstrate a higher rate of chromosome rearrangements in the Capreolinae X chromosome, while the Cervinae X chromosome is more preserved through species radiation. In the subfamily Cervinae, we identify the acrocentric type of cervid X chromosome with the same order of BACs and pericentromeric heterochromatin. This type of X chromosome evolves from the Pecoran ancestral X chromosome (PAX) by one centromere reposition and one inversion. The PAX was reconstructed according to data obtained by X chromosome BAC mapping in species from all artiodactyl families^20^. Variation in X chromosome morphology was observed in Muntiacini tribe due to X-autosomal translocations^10, 38^ and heterochromatin expansion. On the contrary, in the subfamily Capreolinae, we find both types of cervid X chromosome—acrocentric and submetacentric. The European elk (AAL)^20^, reindeer (RTA), Siberian roe deer (CPY) ^20^ and roe deer (*C. capreolus*)^22^ have the submetacentric type of cervid X. In general, the X chromosome order of BAC corresponds to PAX in the studied capreolins, but only the X chromosome of European elk (AAL)^20^ has the ancestral centromere position. In two capreol tribes: Capreolini (Chinese water deer, HIN) and Odocoileini (gray mazama, MGO), we find an acrocentric type of cervid X chromosome. An accumulation of interstitial heterochromatin is also described for the three species: reindeer (RTA), Chinese water deer (HIN), and gray mazama (MGO).

In Capreolinae, the inversion between 93K24/54D24 occurred in paraphyletic groups including water deer (HIN, Capreolini) and gray brocket deer (MGO, Odoloiceini), which may indicate the presence of a common ancestor for these two species. Indeed, the relationships within the Capreolinae subfamily remain controversial, with paraphilias between Capreolini and Odocoileini^39, 40^. However, according to both molecular and morphological data there is no evidence of monophyly for these two species^4^. The occurrence of this inversion in the relatively distant species presents another case of paraphyly within the Capreolinae family. This type of rearrangement is observed in X chromosomes of the subfamily Cervinae. Differences between the acrocentric type of X chromosome in Capreolinae and Cervinae are shown in the whole chromosome alignment map (Fig. 4). In general, the inversion is between 14O10 and 25P8. Inversions in 93K24/54D24 demonstrate the presence of the previously undiscovered breakpoint, which appears in the Cervidae ancestral X chromosome. The alignment demonstrates several major rearrangements consistent with the BAC maps and several new rearrangements not covered by a set of 29 BACs. Overall, the alignment shows that the BAC’s order is well preserved among all five studied cervid X chromosomes, except for several rearrangements, and that the BAC maps are precise and robust.

For ruminants, evolutionary breakpoints^41^ or hot spots of karyotype evolution were identified in previous studies^17^. In addition to the ruminant X chromosome hotspots located between 514O22/316D2, and 108D16/214A3 (108D16/48F6) that were identified in previous studies^20^, rearrangements also occur between 93K24/54D24, with a break in the blue conserved block. Rearrangements at this point have been identified only for Cervidae species, and it must be assumed that this hotspot is specific to this family. In this region, in the cattle genome, there are transcription factors genes, LOC genes (genes with low homology level) and repeated sequences according to previous data^17^.

In rodents, intrachromosomal rearrangements of the X chromosome are associated with large clusters of intrachromosomal duplications and/or repeated DNA sequences which are present in ancestral species but have subsequently disappeared during evolution^42^. We searched for the presence of repeated sequences at cervid X chromosome evolutionary breakpoints and identified genomic coordinates (in Chinese water deer, Chinese muntjac, black muntjac, red deer, and Yarkand deer) of three breakpoints regions (514O22/316D2, 108D16/48F6, and 93K24/54D24) flanking the evolutionary rearrangements (Suppl. 4), and identified repeated sequences in the intervals using RepeatMasker. We identified an increase of LINEs and LTR elements and a decrease of GC-level, SINEs, and DNA element percentages relative to the whole X chromosome only in the breakpoint 108D16/48F6 in all species investigated (Suppl. 5). Further detailed analysis of breakpoint regions is warranted with an additional comparison of the repeatmasked elements in nearby non-breakpoint regions.

## Conclusion

High-resolution X chromosome maps of species in the family Cervidae provide unique information about intrachromosomal evolution and rearrangements. The detailed analysis of the 29 cattle BACs across multiple species by FISH mapping, CDAG staining and bioinformatic analysis allowed us to identify major changes in the course of cervid X chromosome evolution. We detected two types of cervine X-chromosome—acrocentric and submetacentric. The acrocentric type is found in three independent deer linages (subfamily Cervinae, and Capreolinae tribes—Odocoileini and Capreolini). The relationships within Capreolinae subfamily remain controversial, with paraphilias between Capreolini and Odocoileini^39, 40^ tribes. The X chromosome rearrangements identified represent phylogenetic markers that may help to resolve these complicated phylogenetic relationships. According to our data an increase in the rate of X chromosome evolution is observed within the Cervidae family. In ruminants the speed of X chromosome evolution is 1 rearrangement per 15 million years^14^. In the family Cervidae, we find an average rate, including X-autosome translocations, of 2 rearrangements per 10 million years.

## Methods

### Compliance with ethical standards

The study was carried out in compliance with the ARRIVE guidelines. All experiments were approved by the Ethics Committee on Animal and Human Research at the IMCB SB RAS, Russia (No. 01/21 from January 26, 2021), following all relevant guidelines and regulations. This article does not contain any experiments on human subjects performed by any of the coauthors.

### Species

The research was completed using equipment and materials of the Core Facilities Centre “Cryobank of cell cultures” Institute of Molecular and Cellular Biology SB RAS (Novosibirsk, Russia). Cell cultures of black muntjac (*Muntiacus crinifrons*), tufted deer (*Elaphodus cephalophus*) and gray brocket deer (*Mazama gouazoubira*) were provided by Cambridge Resource Center for Comparative Genomics, Cambridge University, UK. Cell cultures of milu deer (*Elaphurus davidianus*) and fallow deer (*Dama dama*) were provided by Laboratory of Genomic Diversity (NCI, Frederick, MD, USA). Cell cultures of sika deer (*Cervus nippon*), red deer (*Cervus elaphus*), Eurasian elk (*Alces alces*), Siberian roe deer (*Capreolus pygargus*) and reindeer (*Rangifer tarandus*) were prepared from ear biopsy at Institute of Molecular and Cellular Biology SB RAS (Novosibirsk, Russia).

### Chromosome preparation

The procedure for establishing the fibroblast cell line from an ear biopsy was described previously^43^. Metaphase chromosomes were obtained from fibroblast cell lines. Briefly, cells were incubated at 37 °C in 5% CO_2_ in medium αMEM (Gibco), supplemented with 15% fetal bovine serum (Gibco), and antibiotics (ampicillin 100 μg/mL, penicillin 100 μg/mL, amphotericin B 2.5 μg/mL). Metaphases were obtained by adding colcemid (0.02 mg/L) and ethidium bromide (1.5 mg/mL) to actively dividing culture for 3–4 h. Hypotonic treatment was performed with 3 mM KCl, 0.7 mM sodium citrate for 20 min at 37 °C and followed by fixation with 3:1 methanol—glacial acetic acid (Carnoy`s) fixative. Metaphase chromosome preparations were made from a suspension of fixed fibroblasts, as described previously^44^. G-banding on metaphase chromosomes prior to fluorescence in-situ hybridization (FISH) was performed using standard procedure^45^. Chromomycin A3-DAPI-after G-banding (CDAG) staining procedure was performed as described earlier^35^.

### FISH procedure

The protocol for selection and coordinates of BAC-clones was reported in previous research^20^. The list of BAC-clones from CHORI-240 library is shown in table (Suppl. 3). BAC clones’ DNA was isolated using the Plasmid DNA Isolation Kit (BioSilica, Novosibirsk, Russia) and amplified with GenomePlex Whole Genome Amplification kit (Sigma-Aldrich Co., St. Louis, MO, USA). Labeling of BAC clone DNA was performed using GenomePlex WGA Reamplification Kit (Sigma-Aldrich Co., St. Louis, MO, USA) by incorporating biotin-16-dUTP or digoxigenin-dUTP (Roche, Basel, Switzerland).

Dual-color FISH experiments were conducted as described by Yang and Graphodatsky^46^. Trypsin-treated chromosomes were immobilized in 0.5% formaldehyde in PBS followed by formamide denaturing and overnight probe hybridization at 40◦C. Digoxigenin-labeled probes were detected using anti-digoxigenin-CyTM3 (Jackson Immunoresearch), whereas biotin-labeled probes were identified with avidin-FITC (Vector Laboratories) and anti-avidin FITC (Vector Laboratories). Images were captured and processed using VideoTesT 2.0 Image Analysis System and a Baumer Optronics CCD Camera mounted on an Olympus BX53 microscope (Olympus).

### Bioinformatic analysis

To perform analysis of chromosome-level assemblies of X, we used available material from GenBank NCBI. Whole X chromosome assemblies were compared with the cattle (NC_037357.1)^37^ X chromosome using D-GENIES^47^ resources by aligner Minimap2 v2.24. After alignment we selected six whole X chromosome assemblies: *Hydropotes inermis* CM035303.1, *Muntiacus reevesi* CM035268.1, *Muntiacus crinifrons* CM018500.1, *Cervus elaphus* OU343077.1, *Cervus hanglu yarkandensis* CM021225.1^31–34^ and *Bos taurus* GK000030.2. After selection we aligned X chromosome assemblies using a whole-genome alignment tool minimap2 2.26-r1175^48, 49^. The search for synteny and structural rearrangements between the X chromosomes was performed using SyRI 1.6.3^50^. For final visualization of intrachromosomal rearrangements plostsr 1.1.0^51^ software was used.

To identify breakpoint coordinates we used aligned in D-GENIES X chromosomes and fixed coordinates. We compared the obtained coordinates with the BAC clones’ coordinates and revealed genome coordinates of intervals (Suppl. 4). Intervals between 514O22/316D2, 108D16/48F6, and 93K24/54D24 were identified. Since only acrocentric chromosomes were used for bioinformatic analysis, instead of the interval 93K24/54D24, which is already broken in these species, the interval 386M2/5424 was used. To identify repeated sequences in a breakpoint interval and in the whole X chromosome of investigated species, RepeatMasker (Dfam 3.2) was used. Visualization of major classes of repeated sequences was performed in Excel (Suppl. 5).

## Supplementary Information

Supplementary Information.

## Data Availability

All data generated or analyzed during this study are included in this article. In research were used data from GenBank NCBI: *Bos taurus* (NC_037357.1, GK000030.2), *Hydropotes inermis* (CM035303.1), *Muntiacus reevesi* (CM035268.1), *Muntiacus crinifrons* (CM018500.1), *Cervus elaphus* (OU343077.1), *Cervus hanglu yarkandensis* (CM021225). Further enquiries can be directed to the corresponding authors.
