# Supplementary material for: Comparative studies of X chromosomes in Cervidae family
Source: Sci Rep. 2023 Jul 25;13:11992. doi: 10.1038/s41598-023-39088-4 (PMC10368622; doi:10.1038/s41598-023-39088-4)
Supplement: Supplementary file 1 — Supplementary Information. [file 41598_2023_39088_MOESM1_ESM.pdf]

Supplementary materials

S1. Schematic summary tree topology of the suborder Ruminantia based on mitochondrial genomes, nuclear genes, and morphological data[1]. The selected branches correspond to species in this study or to closely related species on the phylogeny.

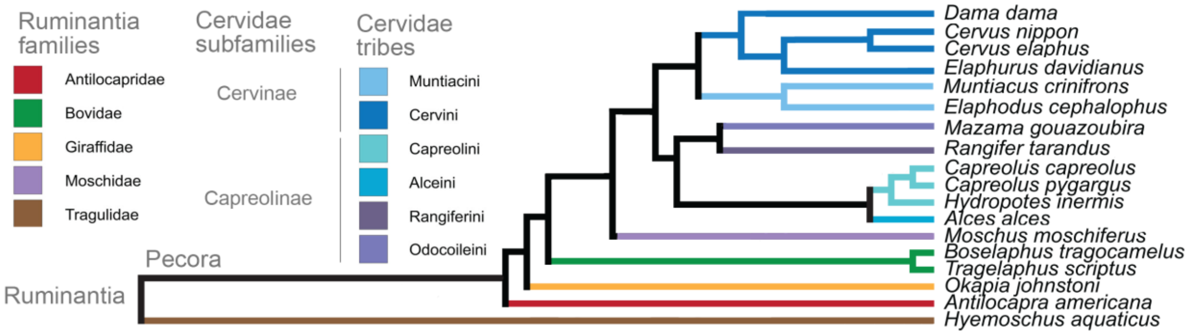

S2. Cervidae X-chromosomes: Capreolinae - Chinese water deer (*Hydropotes inermis*), gray brocket deer (*Mazama gouazoubira*); Cervinae - black muntjac (*Muntiacus crinifrons*), tufted deer (*Elaphodus cephalophus),* sika deer (*Cervus nippon*), and red deer (*Cervus elaphus*) with cattle BACs localization. Centromere positions are designated by a white dot.

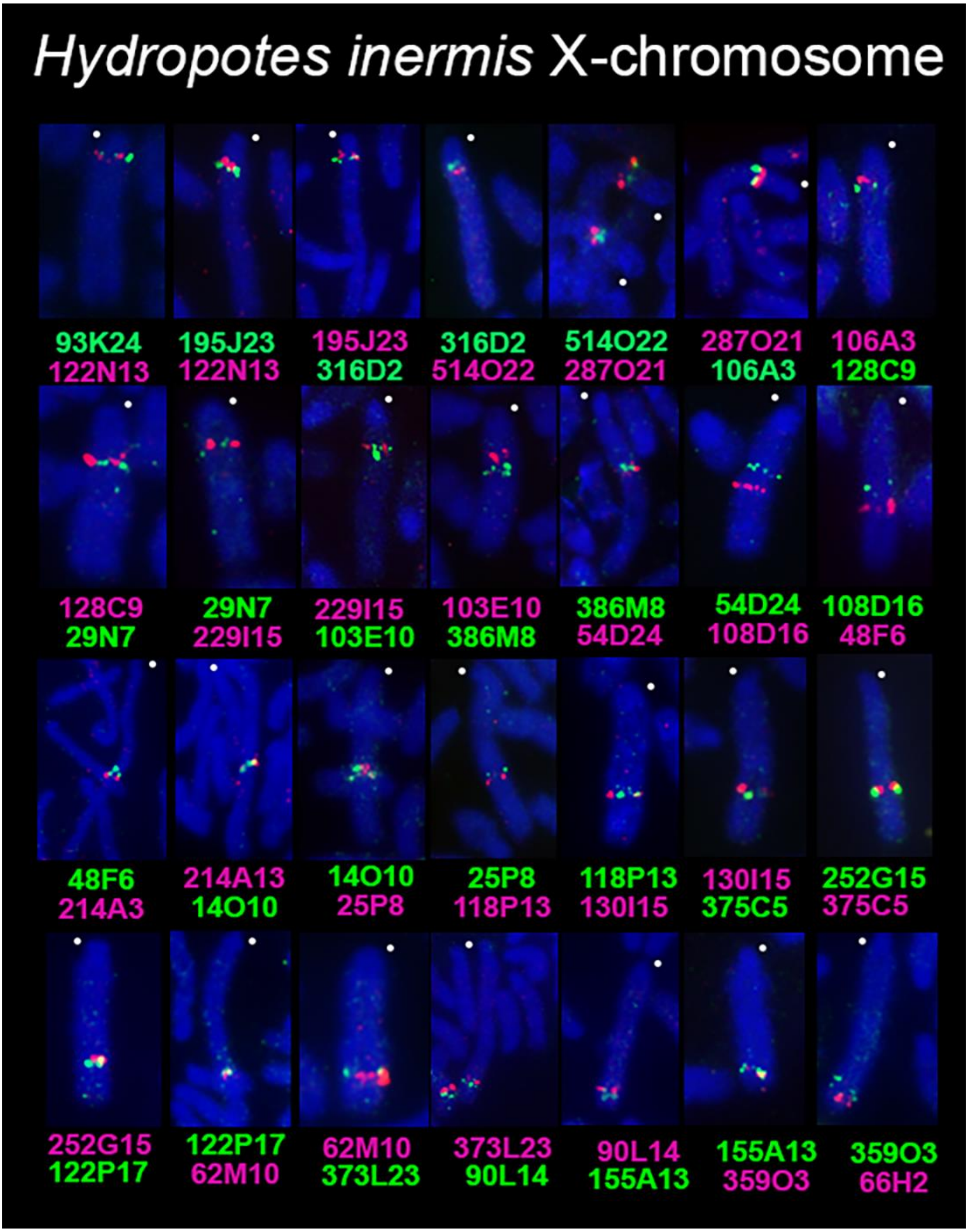

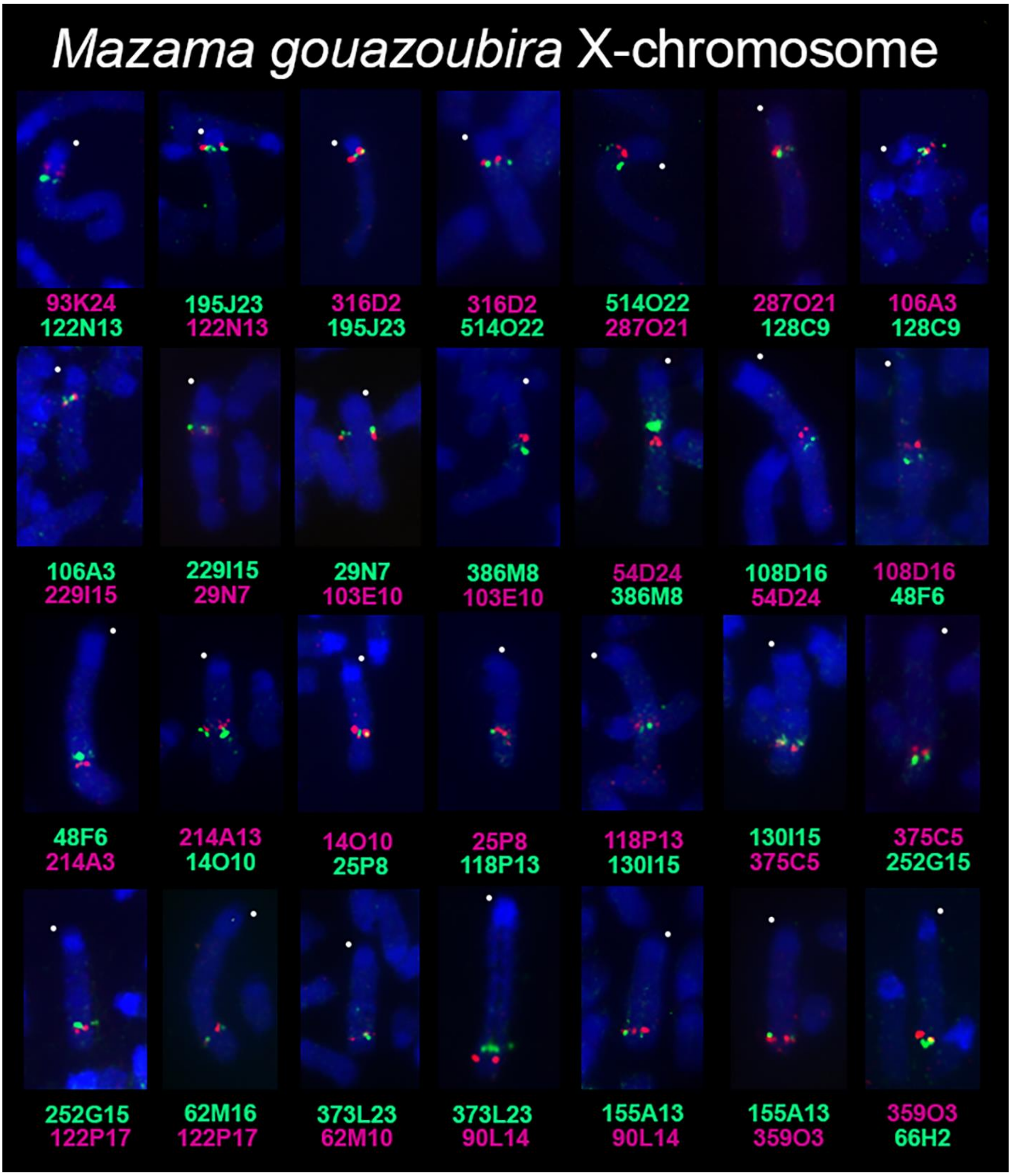
Mazama gouazoubira X-chromosome
93K24
122N13
195J23
122N13
316D2
195J23
316D2
514O22
514O22
287O21
287O21
128C9
106A3
128C9
106A3
229I15
229I15
29N7
29N7
103E10
386M8
103E10
54D24
386M8
108D16
54D24
108D16
48F6
48F6
214A3
214A13
14O10
14O10
25P8
25P8
118P13
118P13
130I15
130I15
375C5
375C5
252G15
252G15
122P17
62M16
122P17
373L23
62M10
373L23
90L14
155A13
90L14
155A13
359O3
359O3
66H2

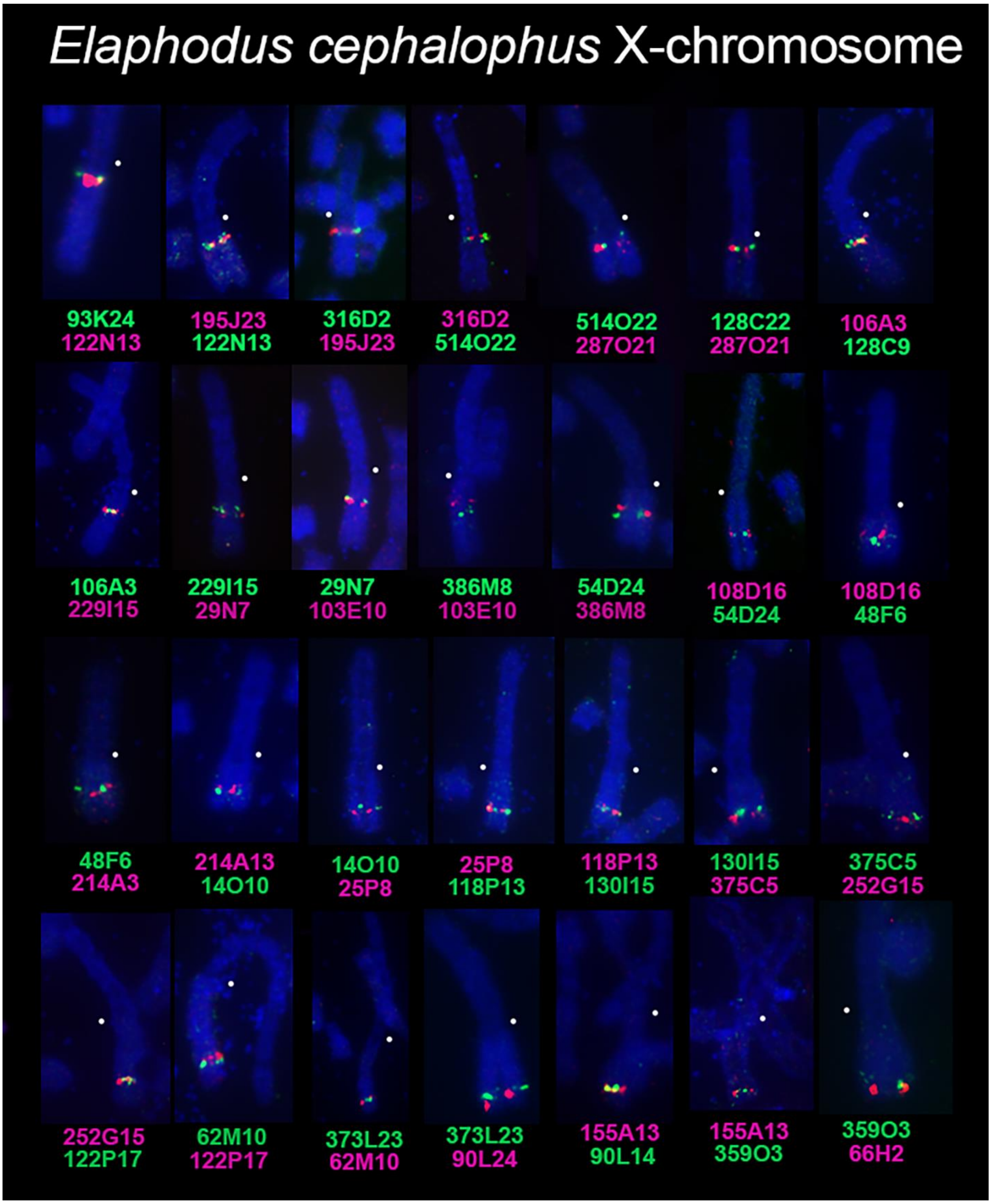
Elaphodus cephalophus X-chromosome
93K24
122N13
195J23
122N13
316D2
195J23
316D2
514O22
514O22
287O21
128C22
287O21
106A3
128C9
106A3
229I15
229I15
29N7
29N7
103E10
386M8
103E10
54D24
386M8
108D16
54D24
108D16
48F6
48F6
214A3
214A13
14O10
14O10
25P8
25P8
118P13
118P13
130I15
130I15
375C5
375C5
252G15
252G15
122P17
62M10
122P17
373L23
62M10
373L23
90L24
155A13
90L14
155A13
359O3
359O3
66H2

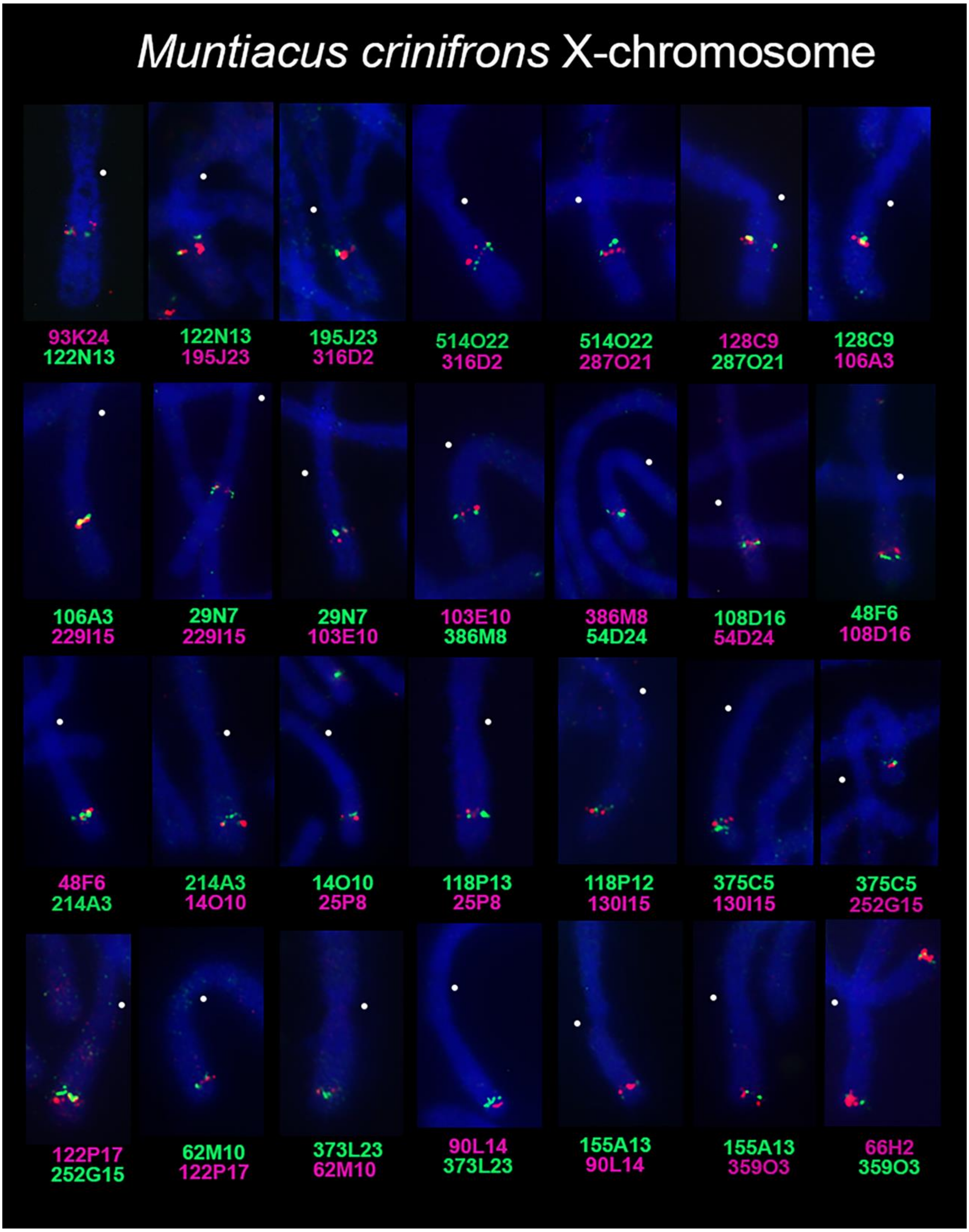
Muntiacus crinifrons X-chromosome
93K24
122N13
122N13
195J23
195J23
316D2
514O22
316D2
514O22
287O21
128C9
287O21
128C9
106A3
106A3
229I15
29N7
229I15
29N7
103E10
103E10
386M8
386M8
54D24
108D16
54D24
48F6
108D16
48F6
214A3
214A3
14O10
14O10
25P8
118P13
25P8
118P12
130I15
375C5
130I15
375C5
252G15
122P17
252G15
62M10
122P17
373L23
62M10
90L14
373L23
155A13
90L14
155A13
359O3
66H2
359O3

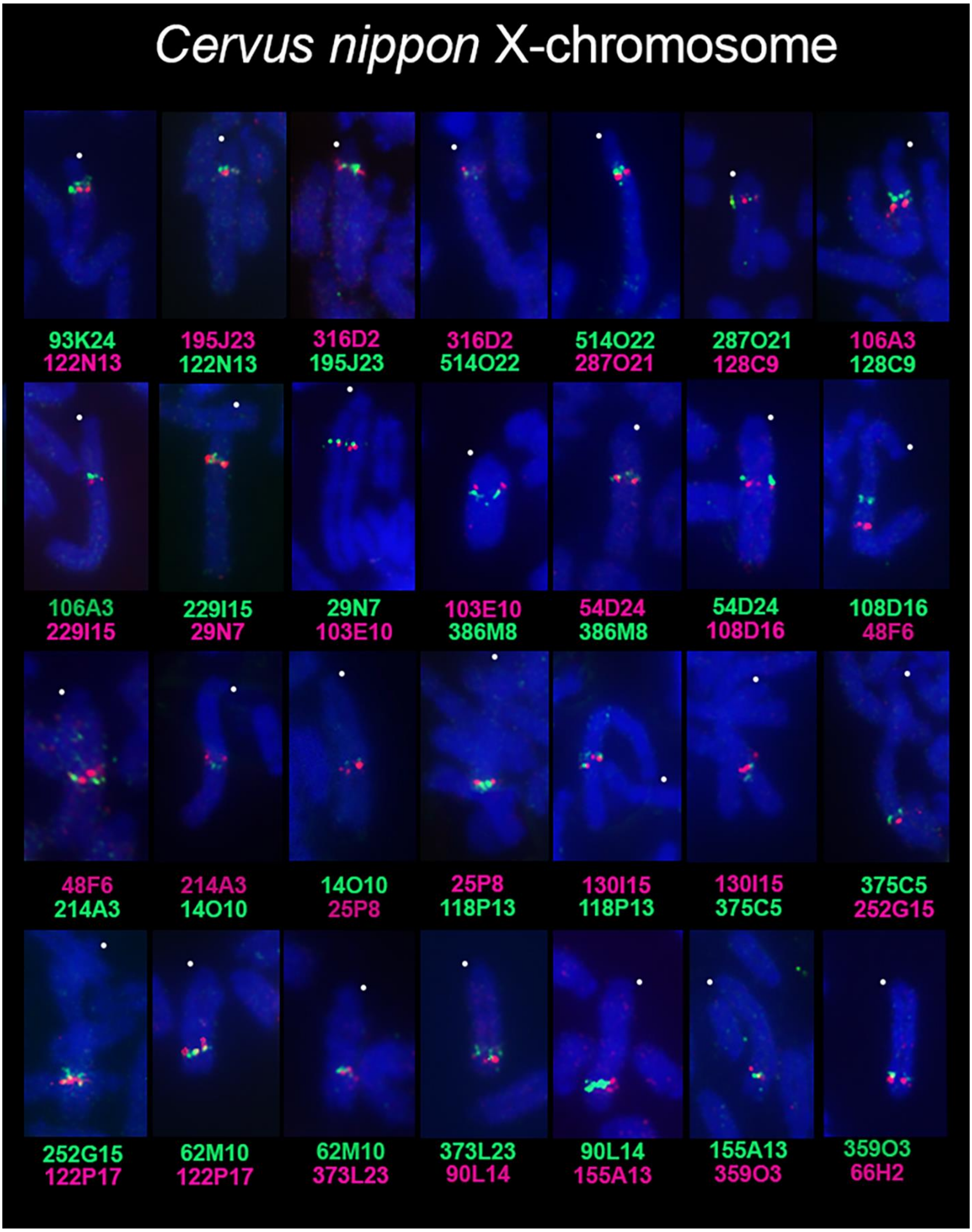
Cervus nippon X-chromosome
93K24
122N13
195J23
122N13
316D2
195J23
316D2
514O22
514O22
287O21
287O21
128C9
106A3
128C9
106A3
229I15
229I15
29N7
29N7
103E10
103E10
386M8
54D24
386M8
54D24
108D16
108D16
48F6
48F6
214A3
214A3
14O10
14O10
25P8
25P8
118P13
130I15
118P13
130I15
375C5
375C5
252G15
252G15
122P17
62M10
122P17
62M10
373L23
373L23
90L14
90L14
155A13
155A13
359O3
359O3
66H2

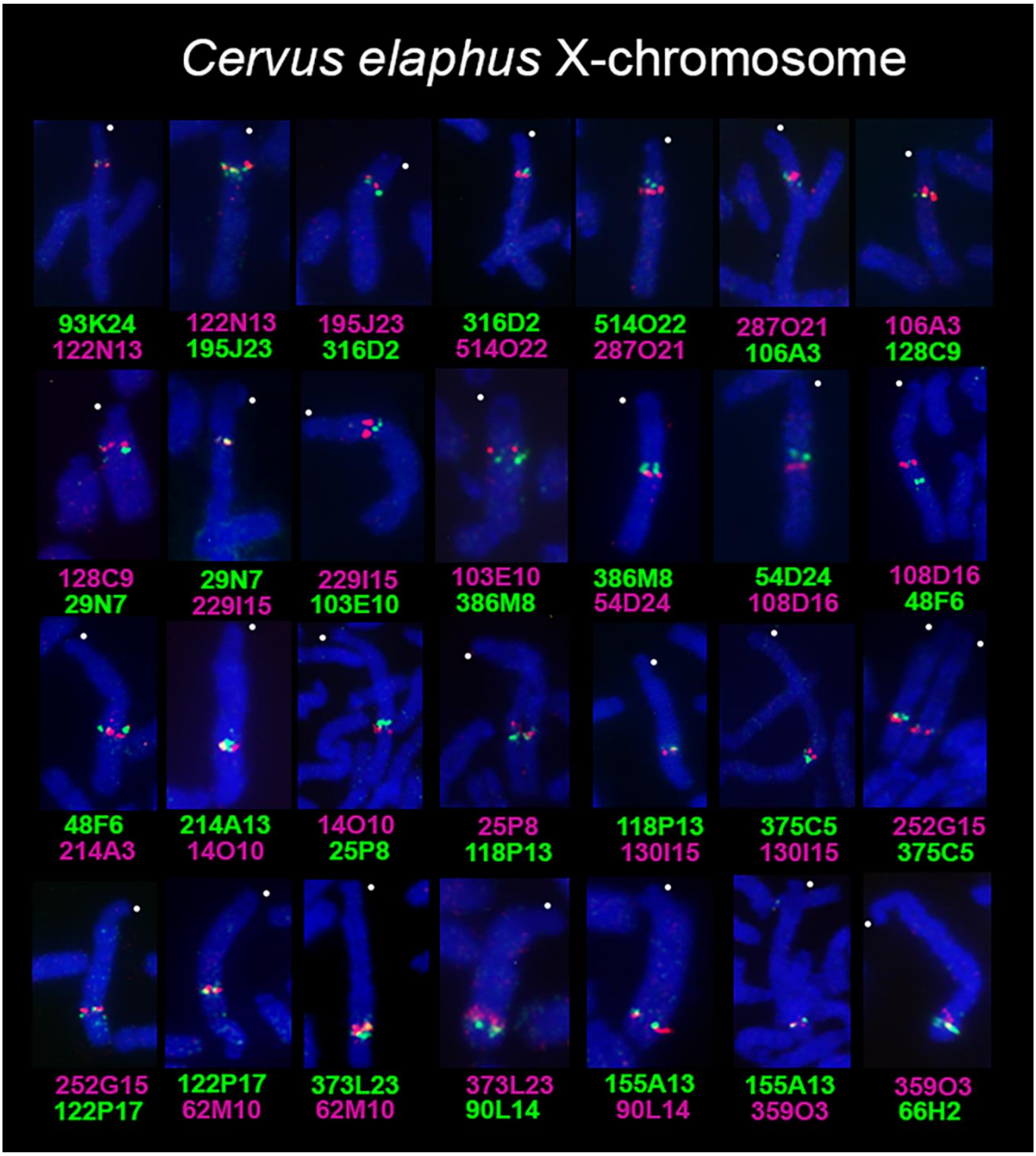

Cervus elaphus X-chromosome
93K24
122N13
122N13
195J23
195J23
316D2
316D2
514O22
514O22
287O21
287O21
106A3
106A3
128C9
128C9
29N7
29N7
229I15
229I15
103E10
103E10
386M8
386M8
54D24
54D24
108D16
108D16
48F6
48F6
214A3
214A13
14O10
14O10
25P8
25P8
118P13
118P13
130I15
375C5
130I15
252G15
375C5
252G15
122P17
122P17
62M10
373L23
62M10
373L23
90L14
155A13
90L14
155A13
359O3
359O3
66H2

S3. CHORI-240 BAC's order on cattle X chromosomes in two genome assemblies. The color of the cells corresponds to a certain conservative syntenic segment.

№	Jun. 2014 (Bos_tautus_UND3.1.1/bosTau8)		Apr. 2018 (ARS-UCD1.2/bosTau9)	
1	514O22	1949353<br>2129088	514O22	2074775<br>2254609
2	287O21	7324034<br>7488466	287O21	7712860<br>7874838
3	128C9	8233624<br>8391009	128C9	8620598<br>8777929
4	106A3	13345128<br>13540519	106A3	13478052<br>13673155
5	229I15	13805346<br>13950311	229I15	13935575<br>14078770
6	29N7	17587835<br>17778937	29N7	17646083<br>17837312
7	103E10	20150516<br>20286170	103E10	Split in new<br>Split in new
8	386M8	33395588<br>33587168	386M8	33202534<br>33394556
9	108D16	48672324<br>48917704	108D16	46121796<br>46365680
10	54D24	53219586<br>53351583	93K24	52414904<br>52626793
11	93K24	57734547<br>57947720	54D24	54441610<br>54574122
12	122N13	62228039<br>62371946	122N13	57786829<br>57930232
13	195J23	62982639<br>63183460	195J23	58524243<br>58725059
14	316D2	68490278<br>68678635	316D2	61306897<br>61316897
15	48F6	79857740<br>80017362	48F6	74820800<br>74979619
16	214A3	84397606<br>84521707	214A3	79103525<br>79227686
17	14O10	85224265<br>85389684	14O10	79926220<br>80093098
18	25P8	90681870<br>90861947	25P8	85417441<br>85598424
19	118P13	92264186<br>92429310	118P13	87185780<br>87358441
20	130I15	95938488<br>96135558	130I15	90539258<br>90737908
21	375C5	103959199<br>104119579	375C5	98546758<br>98707707
22	252G15	108195394<br>108349350	252G15	102735321<br>102889245
23	122P17	110284444<br>110450903	122P17	104942965<br>104954960
24	62M10	111125731<br>111275450	62M10	105646243<br>105796648
25	373L23	117191008<br>117371368	373L23	111893796<br>112073218
26	90L14	126821940<br>127050000	90L14	119874011<br>119903510
27	155A13	128339848<br>128504608	155A13	119989800<br>119991342
28	359O3	128829625<br>128953648	359O3	121586116<br>121710102
29	66H2	141101222<br>141358968	66H2	124117500<br>124118316

S4. Genome intervals of evolutionary breakpoint regions revealed in Chinese water deer (*Hydropotes inermis*), Chinese muntjac (*Muntiacus reevesi*), black muntjac (*Muntiacus crinifrons*), red deer (*Cervus elaphus*), and Yarkand deer (*Cervus hanglu yarkandensis*) X chromosomes.

Name of breakpoint region	Chinese water deer			Chinese muntjac		
	Start	End	Size	Start	End	Size
316D2/514O22	10431271	11037573	606302	123392328	123963467	571139
386M2/54D24	43493793	44824465	1330672	89908314	90164124	255810
108D16/48A6	59958175	62544992	2586817	73153972	75353106	2199134

Name of breakpoint region	Black muntjac			Red deer		
	Start	End	Size	Start	End	Size
316D2/514O22	49622782	49915615	292833	38874812	39348745	473933
386M2/54D24	53497175	53762254	265079	77060107	78778706	1718599
108D16/48A6	87413241	87617034	203793	93886377	96721651	2835274

Name of breakpoint region	Yarkand deer		
	Start	End	Size
316D2/514O22	13068473	13894814	826341
386M2/54D24	45230945	45765345	534400
108D16/48A6	60227031	63139485	2912454

S5. Representativeness of repeated sequences in intervals of identified evolutionary breakpoints and on full X chromosomes of Chinese water deer (*Hydropotes inermis*), Chinese muntjac (*Muntiacus reevesi*), black muntjac (*Muntiacus crinifrons*), red deer (*Cervus elaphus*), and Yarkand deer (*Cervus hanglu yarkandensis*).

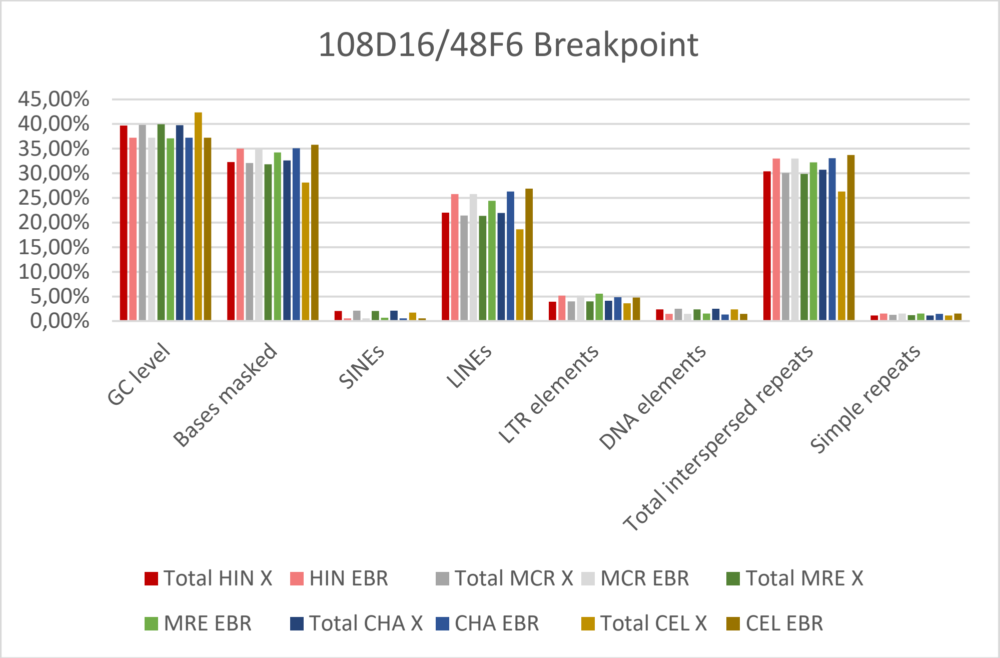

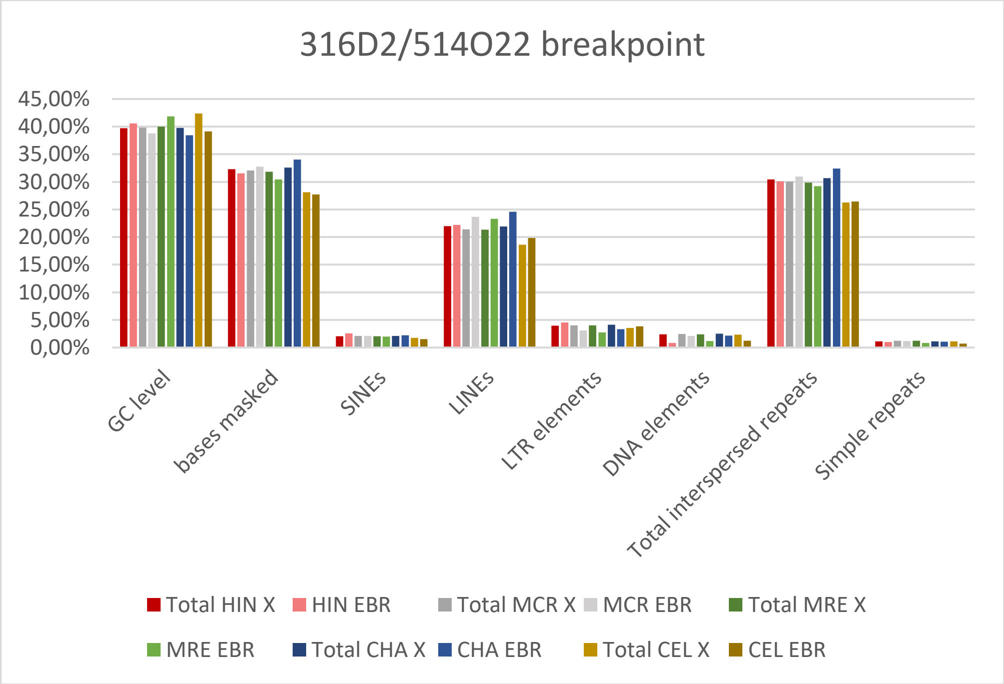

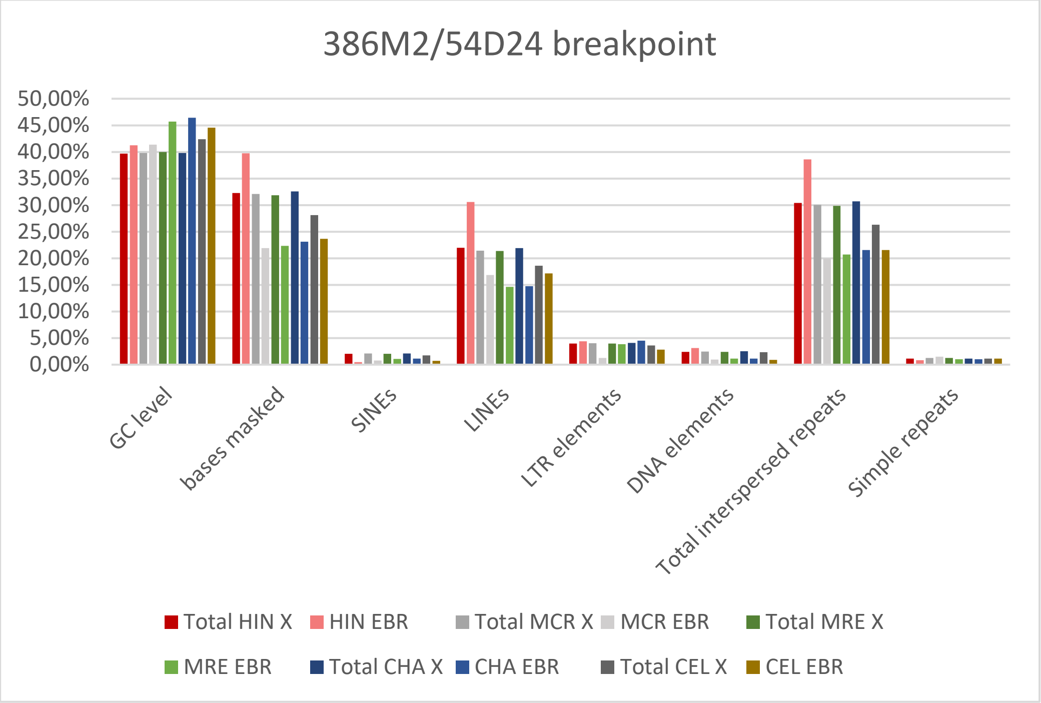

References

1. Heckeberg, N. S. The systematics of the Cervidae: a total evidence approach. *PeerJ* **8**, e8114 (2020).
